# Extraocular Surgical Approach for Placement of Subretinal Implants in Blind Patients: Lessons from Cochlear-Implants

**DOI:** 10.1155/2015/842518

**Published:** 2015-12-10

**Authors:** Assen Koitschev, Katarina Stingl, Karl Ulrich Bartz-Schmidt, Angelika Braun, Florian Gekeler, Udo Greppmaier, Helmut Sachs, Tobias Peters, Barbara Wilhelm, Eberhart Zrenner, Dorothea Besch

**Affiliations:** ^1^Klinikum Stuttgart-Olgahospital, ORL-Department, Pediatric Otorhinolaryngology and Otology, Kriegsbergstraße 62, 70176 Stuttgart, Germany; ^2^Center for Ophthalmology, University of Tübingen, Schleichstraße 12-16, 72076 Tübingen, Germany; ^3^Retina Implant AG, Gerhard-Kindler-Straße 8, 72770 Reutlingen, Germany; ^4^Klinikum Stuttgart-Katharinenhospital, Eye Clinic, Kriegsbergstraße 60, 70174 Stuttgart, Germany; ^5^Klinikum Dresden Friedrichstadt, University Teaching Hospital, Eye Clinic, Friedrichstraße 41, 01067 Dresden, Germany; ^6^STZ Eyetrial, Center for Ophthalmology, University of Tübingen, Schleichstraße 12-16, 72076 Tübingen, Germany; ^7^Werner Reichardt Centre for Integrative Neuroscience (CIN), Otfried-Müller-Straße 25, 72076 Tübingen, Germany

## Abstract

In hereditary retinal diseases photoreceptors progressively degenerate, often causing blindness without therapy being available. Newly developed subretinal implants can substitute functions of photoreceptors. Retina implant extraocular surgical technique relies strongly on cochlear-implant know-how. However, a completely new surgical approach providing safe handling of the photosensor array had to be developed. The Retina Implant Alpha IMS consisting of a subretinal microphotodiode array and cable linked to a cochlear-implant-like ceramic housing was introduced via a retroauricular incision through a subperiosteal tunnel above the zygoma into the orbit using a specially designed trocar. Implant housing was fixed in a bony bed within a tight subperiosteal pocket in all patients. Primary outcomes were patient short term safety as well as effectiveness. Nine patients participated in the first part of the multicenter trial and received the subretinal visual implant in one eye. In all cases microphotodiode array pull-through procedure and stable positioning were possible without affecting the device function. No intraoperative complications were encountered. The minimally invasive suprazygomatic tunneling technique for the sensor unit as well as a subperiosteal pocket fixation of the implant housing provides a safe extraocular implantation approach of a subretinal device with a transcutaneous extracorporeal power supply.

## 1. Introduction

Cochlear-implants (CI) provide hearing restoration replacing the peripheral acoustic receptor by an electronic device. Thus, the idea of bringing vision back to blind patients by replacing the photoreceptive function by technical devices was pursued by a number of groups already since the early nineties [1, 2].

In most hereditary retinal diseases photoreceptors progressively degenerate, often causing blindness in the patient's middle age with no therapy available. The remaining visual pathway remains largely functional [3].

Several types of electronic retinal implants either have been approved as commercial products—Argus II (Second Sight, Sylmar, CA) [2] and Retina Implant Alpha IMS (Retina Implant AG, Reutlingen, Germany) [4]—or are under development for the treatment of hereditary retinal degenerations. All of these implants consist of a light-capturing unit (an external camera or an intraocular photodiode array) and an electrode array for stimulation of retinal neurons, mostly those in the inner retina.

While several groups favor the epiretinal approach [2] with camera outside, our approach was to restore vision by a microelectronic light sensitive device in the subretinal space able to convert light after amplification into electrical signals for driving bipolar cells [1]. This approach makes use of natural eye motility and thereby contributes to more natural visual perception. However the technique of implantation appears more challenging due to the particular location in the subretinal space, which is not regular ophthalmological surgical procedure. Further, the energy supply and parameter settings are transmitted from a small external handheld unit through a receiver coil and electronic circuits in an implant housing similar to cochlear-implants placed in the retroauricular area. Therefore retina implant (RI) extraocular surgical technique relies strongly on CI know-how but had to be newly developed, as the power and signal supply cables had to be brought forward to the orbital area instead of the cochlea.

We describe the extraocular part of the interdisciplinary approach for implantation of a subretinal RI developed by the Tübingen group. This surgical approach was invented during a pilot study in Tübingen [5] and further modified in a following clinical trial [4] leading to the CE-approval of the Alpha IMS (Retina Implant AG, Germany) device in 2013. In the first part of this trial in which this technique was applied the first time, 9 patients were implanted. Eight patients were diagnosed with retinitis pigmentosa and 1 was diagnosed with cone-rod dystrophy. Primary outcomes were patient short term safety (defined as no permanent damage of function and structures that have been functional before surgery) and effectiveness (activities of daily living and mobility significantly improved with implant-ON versus implant-OFF, as shown via tests for simulated activities of daily living, recognition tasks, and mobility) [4]. Meanwhile, altogether 29 patients in 7 centers have been operated on with this technique without any problems in this part of the surgery [4].

## 2. Patients and Methods

### 2.1. Patients

Nine patients (four females, five males) aged 46.9 ± 7.2 years (35–62 years) received the Retina Implant Alpha IMS in one eye in the Center for Ophthalmology, University of Tübingen, Germany, in 2010-2011. Written informed consent in accordance with Declaration of Helsinki was obtained from all subjects prior to inclusion. The study was approved by the local ethics committee and was carried out according to the German Medical Product Law (MPG) and EN ISO 14155 between May 2010 and January 2012. It is registered as NCT01024803 with https://www.clinicaltrials.gov/.

### 2.2. Clinical Characteristics

All 9 patients suffered from a hereditary retinal disease in the end-stage being able to perceive light without correct light source localization (8 patients) or completely blind (no light perception, 1 patient). All patients underwent cataract surgery on the study eye and received an artificial intraocular lens prior to the microchip implantation. Only one eye was implanted. In case of residual light perception differing in the two eyes, the worse one was selected for implantation. For further details, see [4].

All patients were in good general health. The patients reported no serious general diseases or relevant medical history. There were no known contraindications for a general anesthesia.

### 2.3. Device

**Figure 1:**
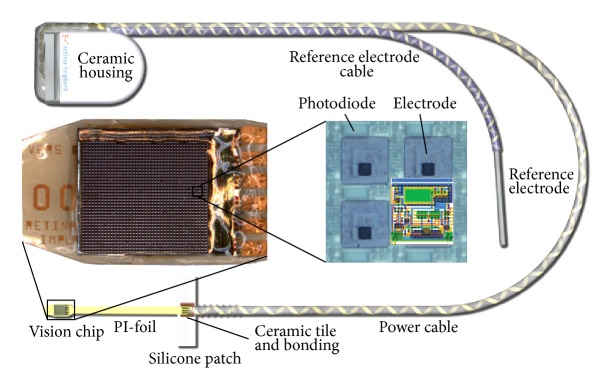
The Retina Implant Alpha IMS consists of the vision chip (multiphotodiode array, 4 pixels magnified in the center) on a polyimide foil (PI-foil, both placed subretinal) and a power supply cable connecting the microchip with the receiver coil in a ceramic housing and the reference electrode placed subdermally at the temple and retroauricular region.

**Figure 2:**
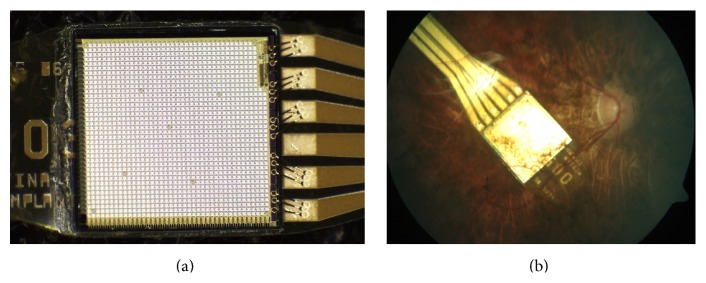
(a) The implant's core is the light sensitive subretinal chip with 1500 pixels. The chip size is approximately 3 mm × 3 mm; it is approximately 70 *μ*m thin when placed on a polyimide foil (thickness approx. 20 *μ*m) with gold connective wire prints, which leaves the subretinal space in the upper temporal periphery through the choroid and the sclera. (b) Fundus image of the Retina Implant Alpha IMS.

**Figure 3:**
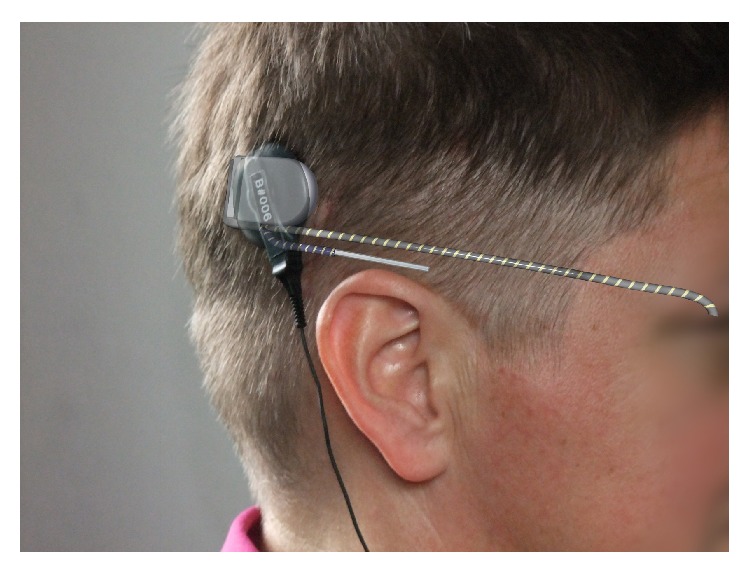
Illustration of the subdermal placement of the receiver coil and the power supply cable (white/grey) in relation to the epidermal transmitter coil (black) and cable leading to the power supply in the patient's pocket.

The implant body contains an electromagnetic receiver coil and amplifier electronics responsible for energy and signal processing. The ceramic housing is similar to the Pulsar cochlear-implant device used in the past by MED-El Company, Innsbruck, Austria. A long cable connects the photosensitive chip to the housing. A second short cable connects the plate of the reference electrode to the implant (Figure 1). The implant's active sensor is a subretinal chip, built from 1500 independent photodiode-amplifier-electrode units, each of which transforms the local luminance information into an electrical current that is amplified for the stimulation of the adjacent bipolar cells (Figure 2). The chip size is approximately 3 mm × 3 mm and 70 *μ*m thin, placed on a stripe of polyimide foil (thickness approx. 20 *μ*m), which leaves the subretinal space in the upper temporal periphery through the choroid and the sclera. The foil is connected to the power supply cable, which, after a loop in the orbit, leads to the retroauricular placed subdermal coil (Figure 3). Here, the inductive transfer of energy and control signals through the skin to the implant is provided via an external transmitter coil powered by a battery pack in the handheld control unit. This battery pack has two knobs for adjusting the amplification and the gain of the amplifiers, thereby adjusting the overall brightness and contrast of the perception according to the particular luminance conditions. This adjustment is performed by the patient after training.

The chip provides the inner retina with a “point-by-point electrical image” of the received luminance information, resulting in an image in grey scales reminiscent of the old black and white TV set. As shown in the clinical trial, Retina Implant Alpha IMS can change blindness into low vision or very low vision in selected patients with hereditary retinal diseases [4]. Chip functional details and the technique of subretinal implantation were described elsewhere [6].

## 3. Surgical Technique and Results

### 3.1. Implantation

**Figure 4:**
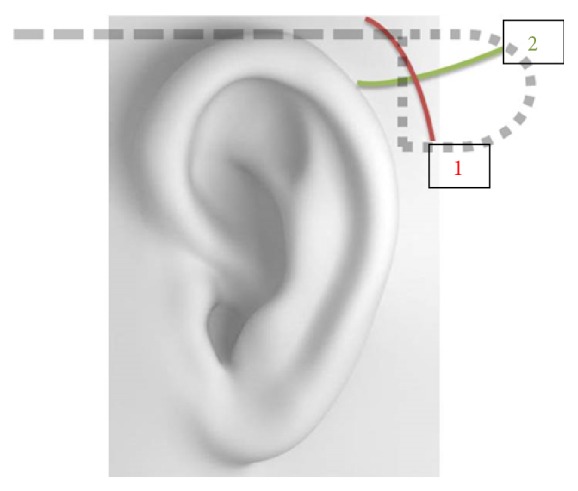
Illustration of the curved retroauricular incision following approximately the helix of the pinna (1) leaving the fascia of the temporal muscle intact. A second horizontal incision (2) was placed along the caudal limit of the temporal muscle crossing nearly perpendicularly the primary skin incision and providing a stabile two-layer wound closure.

The implantation procedure was carried out under general anesthesia. A nonsterile silicone model of the whole implant was used to mark skin incisions and implant position behind the ear and at the orbital rim. After subcutaneous injection of mepivacaine/epinephrine in both regions skin incisions were performed. A curved retroauricular incision was carried out following approximately the helix of the pinna over 4 cm leaving the fascia of the temporal muscle intact (Figure 4). A second horizontal incision was placed along the caudal limit of the temporal muscle crossing nearly perpendicularly the primary skin incision and providing a stabile two-layer wound closure. The periost was elevated using a sharp raspatory beginning from the incision and creating a subperiosteal pocket for the implant housing posteriorly and a tunnel to the infratemporal fossa anteriorly (Figure 4).

A sterile metal model of the implant body was used to mark the bony bed. The depth of the implant bed was 3-4 mm depending on the individual anatomy. Drilling was performed by standard otologic electrical drill (Karl Storz, Tuttlingen, Germany).

**Figure 5:**
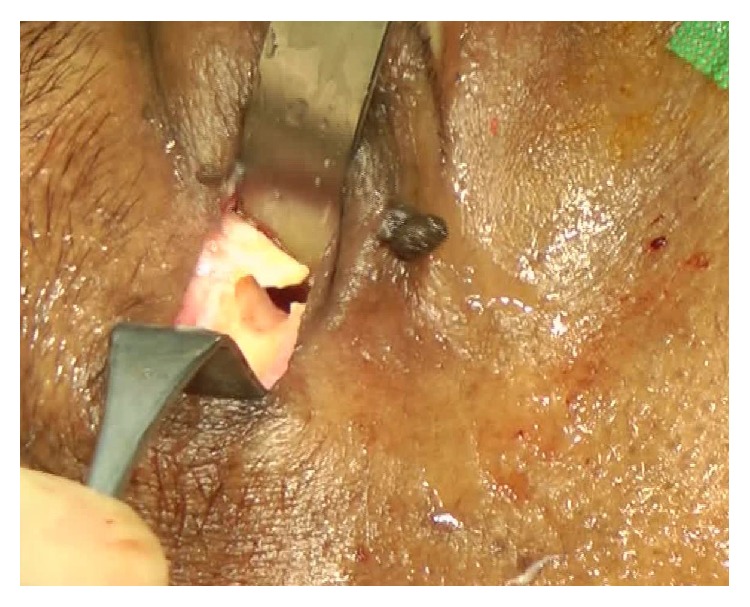
The periost layer was elevated exposing the bone at the sutura frontozygomatica. At this point an L-shaped canal was drilled, providing flexible stability and optimizing angulation for the cable entering the orbit.

The orbital rim incision was placed directly over the margo orbitalis down to the bone. The periost layer was elevated intraorbitally and toward fossa infraorbitalis exposing the bone at the sutura frontozygomatica. At this point an L-shaped canal was drilled (Figure 5), providing flexible stability and optimizing angulation for the cable entering the orbit.

**Figure 6:**
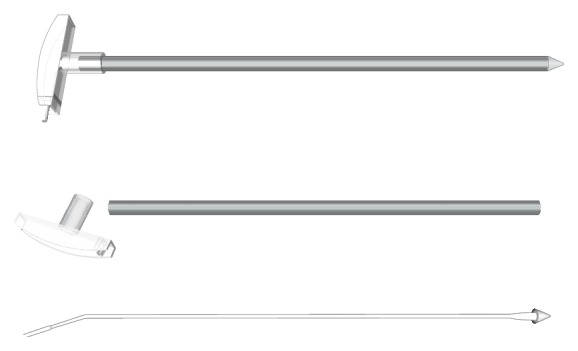
A custom made hollow trocar was advanced from the periorbital region subperiosteally to the retroauricular area. The cone end of the trocar was removed and the sensitive implant chip was placed inside the trocar tube. Retracting the trocar with the implant chip allowed safe passage of the subperiosteal tunnel.

The subperiosteal pocket was connected to the orbital rim incision by tunneling the periost under the temporalis muscle using a long (length 15 cm), slightly curved, sharp raspatory (58210BA, Karl Storz, Tuttlingen, Germany). Following that, a 360° peritomy of the conjunctiva was performed at the limbus. From the subconjunctival space in the upper temporal quadrant, a tunnel was bluntly prepared through the septum to the orbital rim incision and was kept patent by a small silicone tube. A custom made hollow trocar (Figure 6) was advanced from the periorbital region subperiosteally until it reached the retroauricular area. The trocar was introduced directly following and in contact with the end of the raspatory in order to avoid a via falsa and a penetration of periost and temporalis muscle. The cone end of the trocar was removed and the implant chip was then advanced inside the trocar tube. The chip was secured by a silicone sheet filled with physiologic solution. The trocar was extracted by a single movement leading the implant to the orbital rim. The reference electrode remained under the temporalis muscle. The protected implant was pulled through the tunnel to the subconjunctival space and placed aside securely during the following intraocular surgical procedure.

Electrical testing of the implant was performed in all cases at the end of the pull-through procedure to ensure the implant's functionality and integrity.

Wound closure was performed after completion of the intraocular procedure and adjusting the length of the extraocular implant cable.

Extraocular surgery took 60 to 80 minutes, becoming faster according to the learning curve of the team. The extraocular and the intraocular procedures were performed by two different surgical teams.

The intraocular procedure has been described elsewhere in more detail [6]. In short, a scleral flap was prepared in the upper temporal quadrant and the choroid exposed in slit-like manner (approximately 1 mm *∗* 4 mm). The retina was elevated after vitrectomy by subretinal injection of balanced salt solution and/or Healon. The implant was then inserted through the choroid into the subretinal space and forwarded into the desired subfoveal position. Then, the scleral flap and the implant were sutured. The eye was filled with silicone oil as endotamponade after flattening of the retina.

### 3.2. Outcome

There were no intraoperative complications of the extraocular surgery. In all cases the anatomical landmarks were identified and the implant introduction was atraumatic. Electrical testing of the photosensitive chip prior and subsequent to the intraocular procedure revealed normal function in all cases.

During activation of the implant three patients reported pulsing in the area of the temporal muscle. This phenomenon was caused by currents around the reference electrode (placed under the temporal muscle) and was not connected with any relevant discomfort during chip activation. Two patients reported slight tenderness around the coil, probably due to mechanical irritation of the bone scarring, and in none of them was this caused by an inflammation.

In one male patient the thickness of the skin-muscle-layer over the implant housing appeared inappropriate due to more anterosuperior position. Therefore temporalis muscle tissue was reduced. In this patient a diffuse hematoma was observed at the first day after surgery. The hematoma was surgically removed and resolved without sequelae.

Superficial conjunctival hematoma was observed in all patients according to the intraorbital procedures. After approximately 5 days, conjunctival chemosis, edema, and hematomas had almost completely resolved. No serious adverse event was occurring due to the extraocular part of the surgery.

All wounds healed properly, and no signs of infections or wound dehiscence were noticed. Sutures were removed one week after surgery. First activation of the implant was usually performed during the second week after surgery.

During follow-up all extraocular parts of the implant were well tolerated and remained stable in place without migration or extrusion.

Light perception (8/9), light localization (7/9), motion detection (5/9, angular speed up to 35 deg s^−1^), grating acuity measurement (6/9, up to 3.3 cycles per degree), and visual acuity measurement with Landolt C-rings (2/9) up to Snellen visual acuity of 20/546 (corresponding to decimal 0.037 or corresponding to 1.43 logMAR minimum angle of resolution) were restored via the subretinal implant [4]. Additionally, the identification, localization, and discrimination of objects improved significantly (*n* = 8; *p* < 0.05 for each subtest) in repeated tests over a nine-month period. Three subjects were able to read letters spontaneously and one subject was able to read letters after training in an alternative-force choice test. Five subjects reported implant-mediated visual perceptions in daily life within a field of 15° of visual angle [4].

## 4. Discussion

According to our experience the surgical procedure developed for this study is feasible for implantation of a retinal prosthesis with an extraocular implant retroauricular ceramic housing for power supply and control signals. Surgery was uneventful in all cases and led to stable fixation of the implant over the entire study period. The technique of device placement in a periost pocket under the temporalis muscle is well known for cochlear-implants [7, 8]. Due to the implant position of the retina implant behind the pinna a minimally invasive procedure was used. Main orientation points were the helix of the pinna, the ear canal, and the zygomatic process allowing anticipation of the linea temporalis as caudal extension of the temporalis muscle. Due to the limited surgical access the dimension of the subperiosteal pocket fitted exactly the implant body and allowed very tight closure only by suturing the periost without additional fixation over the implant housing.

The intraoperative handling of the delicate structures of the implant appears to be of utmost importance. Since the implant's intra- and extraocular parts are manufactured as a single unit, the device had to be inserted as a whole through the postauricular incision. Further the photosensitive chip had to be pulled through the narrow subperiosteal tunnel in posterior-anterior direction and fixed at the orbital rim. This second point of fixation was performed as an indentation matching exactly the implant cable size. This allows small movements but prevents extrusion. Similar fixation is often used for CI-cable at the mastoid margin.

Passing the chip through the infratemporal tunnel was possible by introducing a specially designed trocar in anterior-posterior direction from orbital rim to the retroauricular incision. This hollow trocar harbored the photosensitive chip and the reference electrode on the way under the temporalis muscle. The introducing of the trocar has to be performed as atraumatic as possible and close to the bone of the infratemporal fossa in order to avoid penetration of the overlaying muscle. Otherwise the implant cable and reference electrode could be misplaced within the temporalis muscle being at risk of permanent tissue movements.

The pulsing around the reference electrode is a side effect of the implant activation with the reference electrode placed under the temporal muscle. To avoid this, currently the reference electrode is placed nearby the ceramic coil. None of the recent patients (not involved in the cohort reported here) reported any pulsing in the region of the temporal muscle afterwards.

The only postoperative complication was a superficial hematoma in one case of untypically anterior implant position, which resolved without sequelae. This was an additional argument for the implant body position behind the pinna, where subcutaneous layer appeared to be most appropriate. Patients felt surprisingly little irritation after the surgical procedure and did not feel uncomfortable with the presence of the device, after the period of wound healing.

## 5. Conclusions

The minimally invasive suprazygomatic tunneling technique for the sensor unit and a subperiosteal pocket fixation of the implant housing provides a safe extraocular implantation approach of a subretinal device with a transcutaneous extracorporeal energy source.

The extraocular part of the implantation procedure was performed atraumatically allowing integrity of the photosensitive chip in all cases. During the implantation period implant migration was not observed.

Meanwhile, the extraocular procedure, developed and described here, has been adopted and used successfully by seven additional centers in the ongoing multicenter trial with the Alpha IMS device [9].
